# Supplementary material for: AT101 [(-)-Gossypol] Selectively Inhibits MCL1 and Sensitizes Carcinoma to BH3 Mimetics by Inducing and Stabilizing NOXA
Source: Cancers (Basel). 2020 Aug 15;12(8):2298. doi: 10.3390/cancers12082298 (PMC7464284; doi:10.3390/cancers12082298)

AT101 [(-)-Gossypol] Selectively Inhibits MCL1 and Sensitizes Carcinoma to BH3 Mimetics by Inducing and Stabilizing NOXA

David J. Mallick and Alan Eastman

Supplemental Protocols and Figures

Table S1
Checklist MIQE

ITEM TO CHECK	IMPORTANCE	CHECKLIST/EXPLANATION
**EXPERIMENTAL DESIGN**		
Definition of experimental and control groups	**E**	**Fig. 1C - Experimental:** Cells incubated with either 10 μM AT101, 1 μM BNC105, 20 μM S1 and 30 μM UMI-77 for 6 h. **Control:** Untreated cells; **Fig. 2B and 2G - Experimental:** HCT116 cells incubated with 0-10 μM AT101 for 6 h. **Control:** Untreated HCT116 cells; **Fig. 2E - Experimental:** HCT116 cells incubated with intracellular Ca^{2+} chelator BAPTA AM for 1 h then 0-10 μM AT101 was added for 6 h. **Control:** Untreated HCT116 cells; **Fig. 5B - Experimental:** HCT116 cells incubated with 10 μM AT101 for 6 h, then washed and incubated for a further 6-48 h in complete media only. **Control:** Untreated HCT116 cells.
Number within each group	**E**	**Fig. 1C:** n=3 for all groups; **Fig. 2B and 2G:** n=3 for all groups; **Fig. 2E:** n=3 for all groups; **Fig. 5B:** n=3 for all groups.
Assay carried out by core lab or investigator's lab?	D	Assays carried out by investigator's laboratory.
Acknowledgement of authors' contributions	D	Dr. David Mallick performed all qPCR experiments in this paper. Acknowledgements are made on lines 494-495.
**SAMPLE**		
Description	**E**	All experimental samples were generated from cell line cultures that were trypsinized (except suspension cultures) and washed in 1x PBS prior to RNA isolation. **Fig. 1C - Experimental samples:** adherent or suspended cells from cell lines incubated with 10 μM AT101, 1 μM BNC105, 20 μM S1 or 30 μM UMI-77 for 6 h. **Control samples:** adherent or suspended cells from untreated cell lines; **Fig. 2B and 2G - Experimental samples:** adherent HCT116 cells incubated with 0-10 μM AT101 for 6 h. **Control samples:** untreated adherent HCT116 cells; **Fig. 2E - Experimental samples:** adherent HCT116 cells incubated with intracellular Ca2+ chelator BAPTA AM for 1 h then 0-10 μM AT101 was added for 6 h. **Control samples:** untreated adherent HCT116 cells; **Fig. 5B - Experimental samples:** adherent HCT116 cells incubated with 10 μM AT101 for 6 h, then washed and incubated for a further 6-48 h in complete media only. **Control samples:** untreated adherent HCT116 cells.
Volume/mass of sample processed	D	Cells were processed for RNA isolation in 100 μL of 1x PBS.
Microdissection or macrodissection	**E**	not applicable
Processing procedure	**E**	**Applicable to all assays:** After cell lines were incubated with the indicated compounds, cells were trypsinized using 0.05% trypsin solution then resuspended in cold, 1x PBS. Cells were then pelleted at 12000 *g* for 30 s, resuspended in 100 μL 1x PBS, then pelleted at 12000 *g* for 5 min. Samples were then immediately processed through our RNA isolation protocol. For long-term storage, after cells were pelleted at 12000 *g* for 30 s, the pellets were resuspended in a 5:1 volume solution containing RNA*later*™ Stabilization solution and 1x PBS and stored in 4 °C for up to one week.
If frozen - how and how quickly?	**E**	Not frozen.
If fixed - with what, how quickly?	**E**	Not fixed.
Sample storage conditions and duration (especially for FFPE samples)	**E**	If required, samples were stored in RNA*later*™ Stabilization solution and 1x PBS in 4°C for up to one week.
**NUCLEIC ACID EXTRACTION**		
Procedure and/or instrumentation	**E**	Total mRNA was extracted using TRIzol Reagent (Sigma-Aldrich) according to the manufacturer's protocol. Homogenization of samples was performed in 1.5 mL microcentrifuge tubes using 1 mL of TRIzol reagent per $0.5x10^6$ cells. Cell lysates were passed through a pipette several times and the homogenate was stored for 5 min at room temperature (RT). Following homogenization, 0.1 mL of 1-bromo-3-chloropropane (BCP) was added and the samples were vortexed for 15 seconds. Samples were then stored for 15 min at RT, followed by centrifugation at 12,000 *g* for 15 min at 4 °C. The aqueous phase of each sample was then transferred to a clean tube and 0.5 mL isopropanol was added. Samples were inverted to mix and stored for 5-10 min at RT. Samples were then centrifuged at 12,000 *g* for 8 min at 4 °C to pellet RNA. Excess liquid was removed and RNA pellets were twice washed in 1 mL of 75% ethanol and centrifuged at 7,500 *g* for 5 min at 4 °C. Following washes, excess liquid was removed and RNA pellets were air-dried for 5-10 min. RNA pellets were then dissolved in 50 μL Molecular Grade Water and stored at -80 °C.
Name of kit and details of any modifications	**E**	TRIzol Reagent (followed manufacturer's protocol)
Source of additional reagents used	D	1-bromo-3-chloropropane (BCP): Molecular Research Center, Inc.; Isopropanol: Fisher Scientific; Ethanol: Fisher Scientific; Molecular Grade Water: Bio-Rad
Details of DNase or RNAse treatment	**E**	No Dnase or RNAse treatment performed.
Contamination assessment (DNA or RNA)	**E**	Contamination of RNA samples was assessed through measurement of the A260/280 ratio on a NanoDrop Spectrophotometer. A ratio value above 1.8 was considered to be of acceptable purity (i.e. very little DNA or protein contaminiation).
Nucleic acid quantification	**E**	The RNA concentration of each sample was determined by measuring the absorbance at 260 nm on a NanoDrop Spectrophotometer.
Instrument and method	**E**	NanoDrop 200 Spectrophotometer-1 (Bio-Rad)
Purity (A260/A280)	D	RNA purity was determined by measuring the A260/280 ratio.
Yield	D	RNA yield for cultured cells was around 8-15 μg per 10^6 cells
RNA integrity method/instrument	**E**	No RNA integrity method/instrument was used.
RIN/RQI or Cq of 3' and 5' transcripts	**E**	Not applicable.
Electrophoresis traces	D	Not applicable.
Inhibition testing (Cq dilutions, spike or other)	**E**	No inhibition testing was performed.

**REVERSE TRANSCRIPTION**		
Complete reaction conditions	**E**	**Reverse transcription of mRNA:** cDNA was synthesized from mRNA using iScript™ cDNA Synthesis Kit (Bio-Rad) according to the manufacturer's protocol. All samples were prepared in 200 µL qPCR tubes. Approximately 400 ng of RNA was mixed with 5 µL of 5x iScript Reaction Mix and Reverse Transcriptase (4:1 ratio of Reaction Mix to Reverse Transcriptase) and diluted up to 20 µL with Nuclease-free water. Each sample was vortexed for <5 seconds and stored on ice. All samples were then incubated in a Veriti 96-Well Programmable Thermocycler at the following settings: 5 min at 25 °C for priming, 30 min at 42 °C for reverse transcription to occur, 5 min at 85 °C for inactivation of reverse transcriptase. All samples were then held at 4 °C until taken off the thermocycler. A No-RT control sample was created at this step in which RNA from the NT sample was mixed with 5x iScript Reaction Mix containing no Reverse Transcriptase.
Amount of RNA and reaction volume	**E**	400 ng of RNA; 20 µL of volume per sample
Priming oligonucleotide (if using GSP) and concentration	**E**	Did not use GSP.
Reverse transcriptase and concentration	**E**	RNase H+, 10 units/µL
Temperature and time	**E**	Specified in "Complete reaction conditions"
Manufacturer of reagents and catalogue numbers	D	Bio-Rad (Cat# 1708890)
Cqs with and without RT	D*	In all experiments, no Cq was achieved for samples without RT compared to samples with RT.
Storage conditions of cDNA	D	All cDNA samples were stored at -20 °C.
**qPCR TARGET INFORMATION**		
If multiplex, efficiency and LOD of each assay.	**E**	Not applicable.
Sequence accession number	**E**	**PMAIP1 (NOXA):** NM_001382617; **MCL1:** NM_021960; **GAPDH:** NM_001289745
Location of amplicon	D	Not applicable.
Amplicon length	**E**	**PMAIP1 (NOXA):** 279 nt; **MCL1:** 210 nt; **GAPDH:** 449 nt
In silico specificity screen (BLAST, etc)	**E**	Specificity was determined by BLAST. **PMAIP1 (NOXA):** score = 46.1 bits; **MCL1:** score = 46.1 bits; **GAPDH:** score = 46.1 bits
Pseudogenes, retropseudogenes or other homologs?	D	No pseudogenes, retropseudogenes or other homologs were used.
Sequence alignment	D	**MCL1:** 790 to 810 (100% identities); **GAPDH:** 159 to 181 (100% identities)
Secondary structure analysis of amplicon	D	No secondary structure analysis was applied.
Location of each primer by exon or intron (if applicable)	**E**	Not applicable.
What splice variants are targeted?	**E**	No splice variants were targeted.
**qPCR OLIGONUCLEOTIDES**		
Primer sequences	**E**	Listed in paper under "RT-qPCR Analysis"
RTPrimerDB Identification Number	D	Not available.
Probe sequences	D**	Not applicable.
Location and identity of any modifications	**E**	Not applicable.
Manufacturer of oligonucleotides	D	Integrated DNA Technologies (IDT)
Purification method	D	Standard Desalting
**qPCR PROTOCOL**		
Complete reaction conditions	**E**	RT-qPCR reactions were performed in a 25 µL reaction containing 0.5 µL of diluted cDNA, 12.5 µL 2x EvaGreen MasterMix and 0.5 µL of each forward and reverse primer at final concentrations of 200 nM. All qPCR reactions were run in triplicate using TempAssure® 0.2 mL PCR 8-tube strips and optical caps (USA Scientific, Ocala, FL) on a CFX96 Real-Time System (Bio-Rad, Hercules, CA). Amplification conditions were adapted to each primer based on their primer melting temperature (Tm), but mainly consisted of the initial denaturation step at 95.0 °C for 10 min, followed by approximately 40 cycles of 30 s at 94.0 °C, primer melting temperature for 30 s and 72.0 °C for 1 min. Afterwards, a melting curve was generated for each primer set to confirm the presence of a single uniform peak, which indicates on-target primer efficiency.
Reaction volume and amount of cDNA/DNA	**E**	Reaction volume: 25 µL; Amount of cDNA: 1.7 µL of a 1/20 dilution
Primer, (probe), Mg++ and dNTP concentrations	**E**	200 nM primers (forward and reverse), 1x EvaGreen qPCR MasterMix, 0.2 mM dNTP, 3mM $MgCl_2$
Polymerase identity and concentration	**E**	Cheetah™ hot-start Taq polymerase (Biotium; Cat# 29050) at a final concentration of 0.02 units/µL
Buffer/kit identity and manufacturer	**E**	2x PCR Buffer (Biotium) containing Tris and $MgCl_2$)
Exact chemical constitution of the buffer	D	Biotium provided the buffer but exact composition is not available.
Additives (SYBR Green I, DMSO, etc.)	**E**	None. All ingredients in master mix, including EvaGreen
Manufacturer of plates/tubes and catalog number	D	TempAssure® 0.2 mL PCR 8-tube strips and optical caps (USA Scientific, Ocala, FL); Cat# 1402-2680
Complete thermocycling parameters	**E**	Initial denaturation step at 95.0 °C for 10 min, followed by approximately 40 cycles of 30 s at 94.0 °C, primer melting temperature for 30 s at 72.0 °C for 1 min.
Reaction setup (manual/robotic)	D	Manual
Manufacturer of qPCR instrument	**E**	CFX96 Real-Time System (Bio-Rad)
**qPCR VALIDATION**		
Evidence of optimization (from gradients)	D	No evidence available.
Specificity (gel, sequence, melt, or digest)	**E**	A melting curve was generated for each primer set to confirm the presence of a single uniform peak, which indicates on-target primer efficiency.
For SYBR Green I, Cq of the NTC	**E**	EvaGreen was used for quantitation. Using EvaGreen, the NTC sample never reached the threshold signal for all qPCR experiments after 40 cycles. Therefore the was no Cq value assigned to the NTC control.
Standard curves with slope and y-intercept	**E**	No standard curves performed.
PCR efficiency calculated from slope	**E**	No PCR efficiency calculated.
Confidence interval for PCR efficiency or standard error	D	Not applicable.
r2 of standard curve	**E**	Not available.
Linear dynamic range	**E**	Not available.
Cq variation at lower limit	**E**	Not available.
Confidence intervals throughout range	D	Not applicable.
Evidence for limit of detection	**E**	Not available.
If multiplex, efficiency and LOD of each assay.	**E**	Not applicable.

DATA ANALYSIS		
qPCR analysis program (source, version)	**E**	CFX Manager Software™
Cq method determination	**E**	The threshold is determined using the Amplification-based threshold method. The threshold is used to determine the Cq values of all samples.
Outlier identification and disposition	**E**	A Cq value was discarded based if its corresponding melting curve was found to be inconsistent with out samples and controls in the assay. For all assays in this study, no Cq values were discarded.
Results of NTCs	**E**	For EvaGreen, the NTC sample never reached the threshold signal for all qPCR experiments after 40 cycles. Therefore the was no Cq value assigned to the NTC control.
Justification of number and choice of reference genes	**E**	Our reference gene choice for all qPCR assays performed in this study was GAPDH. GAPDH is a standard housekeeping gene whose expression was found to be unaffected by the compounds and incubation conditions used in this study.
Description of normalization method	**E**	Normalization method is described in the text (under "RT-qPCR Analysis").
Number and concordance of biological replicates	D	There were three biological replicates performed for all qPCR experiments in this study.
Number and stage (RT or qPCR) of technical replicates	**E**	All qPCR reactions were performed in technical triplicate (in addition to biological replicates)
Repeatability (intra-assay variation)	**E**	Mean standard deviation of triplicates for all assays: 0.10 - 0.30
Reproducibility (inter-assay variation, %CV)	D	Mean standard deviation of biological triplicates: **Fig. 1C:** 0.39 (HCT116), 0.43 (SW620), 0.30 (MiaPaca2), 0.27 (MDA-MB-231), 0.37 (PC3), 0.42 (NB4); **Fig. 2B:** 0.30; **Fig. 2G:** 0.30; **Fig. 2E:** 0.32; **Fig. 5B:** 0.34
Power analysis	D	No power analysis performed.
Statistical methods for result significance	**E**	The unpaired, two-tailed Student's *t*-test was applied to the dC(t) values to determine the statistical significance between experiment groups.
Software (source, version)	**E**	Microsoft Excel (Version 16.39); GraphPad Prism (Version 8.4.3)
Cq or raw data submission using RDML	D	Not available.

Table 1. MIQE checklist for authors, reviewers and editors. All essential information (E) must be submitted with the manuscript. Desirable information (D) should be submitted if available. If using primers obtained from RTPrimerDB, information on qPCR target, oligonucleotides, protocols and validation is available from that source.

*: Assessing the absence of DNA using a no RT assay is essential when first extracting RNA. Once the sample has been validated as RDNA-free, inclusion of a no-RT control is desirable, but no longer essential.

**: Disclosure of the probe sequence is highly desirable and strongly encouraged. However, since not all commercial pre-designed assay vendors provide this information, it cannot be an essential requirement. Use of such assays is advised against.

Uncropped western blots

Immunoblot analysis: Following transfer of proteins to a PVDF membrane, the membrane was cut into sections appropriate for the size of each antigen. This permits several antigens to be analyzed from the same membrane and reduces the amount and volume of antibody required. After incubation with the primary antibody, each membrane is incubated with a fluorescent secondary antibody. Finally, the membrane is scanned on the fluorescent imager and the image analysis provides direct quantification of each band over a large dynamic range.

Quantification of all bands is presented as a percentage of the untreated control on each blot. In the case of PARP, the results are presented both as the relative amount of the parent band, as well as the percent cleaved (i.e., cleaved/total). The area used for the figures in the paper are outlined by a yellow box.

Figure 2I is an exception in that it used enhanced chemiluminescence detection, and quantitation was obtained by densitometry.

Figure 1A

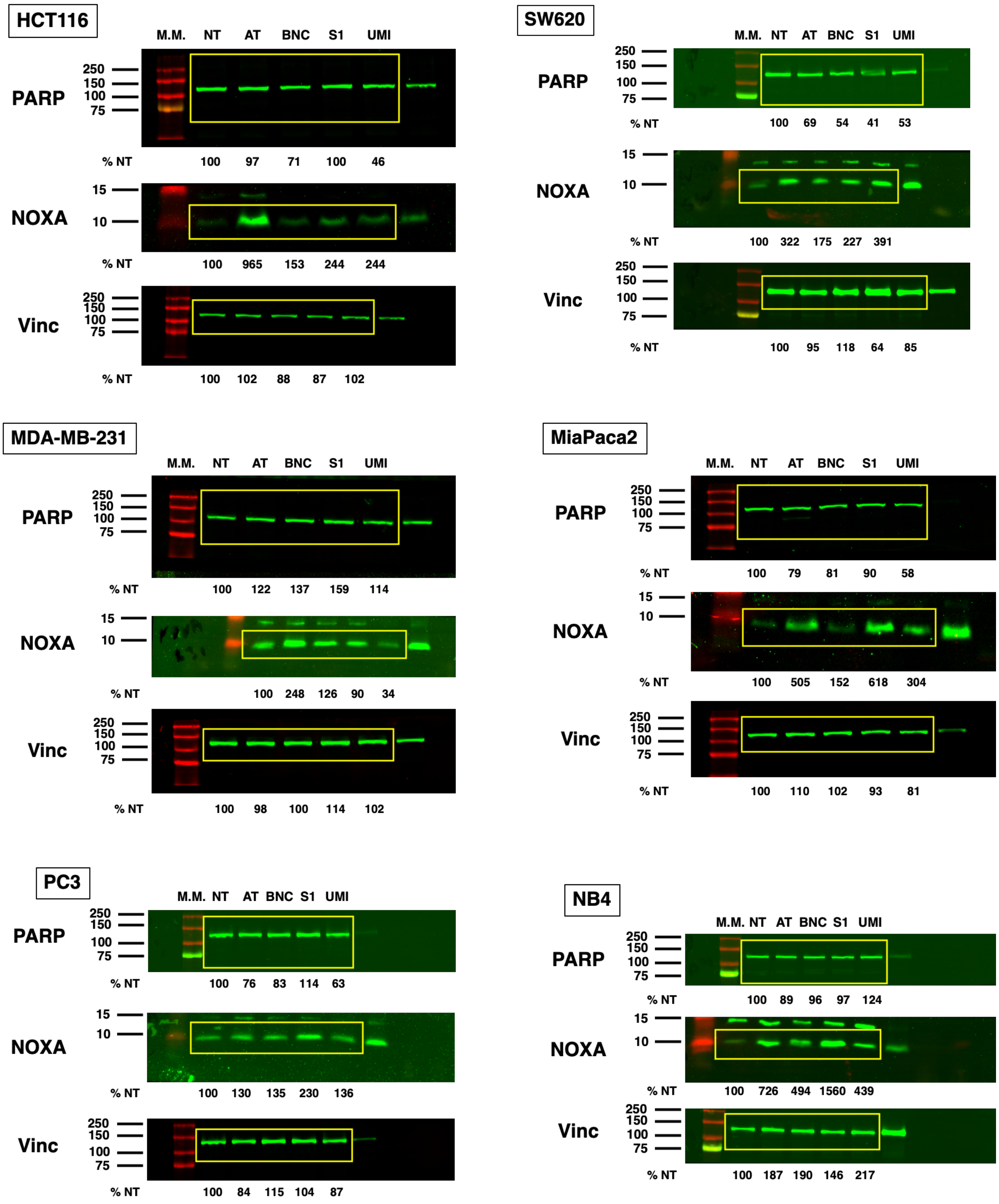
HCT116
M.M. NT AT BNC S1 UMI
PARP
250
150
100
75
% NT 100 97 71 100 46
NOXA
15
10
% NT 100 965 153 244 244
Vinc
250
150
100
75
% NT 100 102 88 87 102
SW620
M.M. NT AT BNC S1 UMI
PARP
250
150
100
75
% NT 100 69 54 41 53
NOXA
15
10
% NT 100 322 175 227 391
Vinc
250
150
100
75
% NT 100 95 118 64 85
MDA-MB-231
M.M. NT AT BNC S1 UMI
PARP
250
150
100
75
% NT 100 122 137 159 114
NOXA
15
10
% NT 100 248 126 90 34
Vinc
250
150
100
75
% NT 100 98 100 114 102
MiaPaca2
M.M. NT AT BNC S1 UMI
PARP
250
150
100
75
% NT 100 79 81 90 58
NOXA
15
10
% NT 100 505 152 618 304
Vinc
250
150
100
75
% NT 100 110 102 93 81
PC3
M.M. NT AT BNC S1 UMI
PARP
250
150
100
75
% NT 100 76 83 114 63
NOXA
15
10
% NT 100 130 135 230 136
Vinc
250
150
100
75
% NT 100 84 115 104 87
NB4
M.M. NT AT BNC S1 UMI
PARP
250
150
100
75
% NT 100 89 96 97 124
NOXA
15
10
% NT 100 726 494 1560 439
Vinc
250
150
100
75
% NT 100 187 190 146 217

Figure 2A

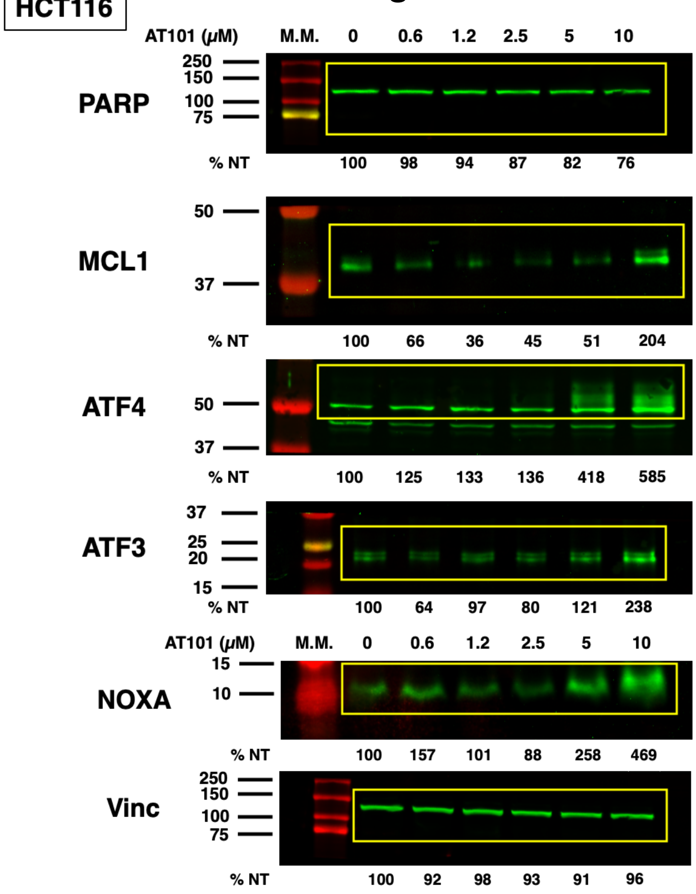

Figure 2C

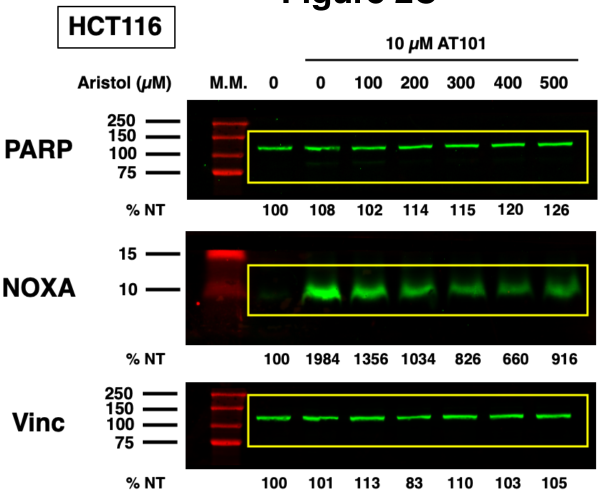

Figure 2D

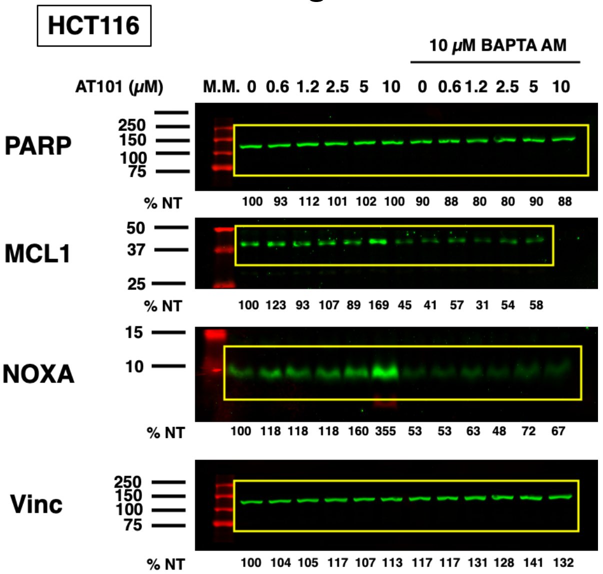

Figure 2F

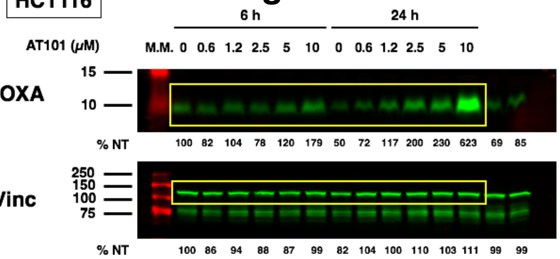

Figure 2G

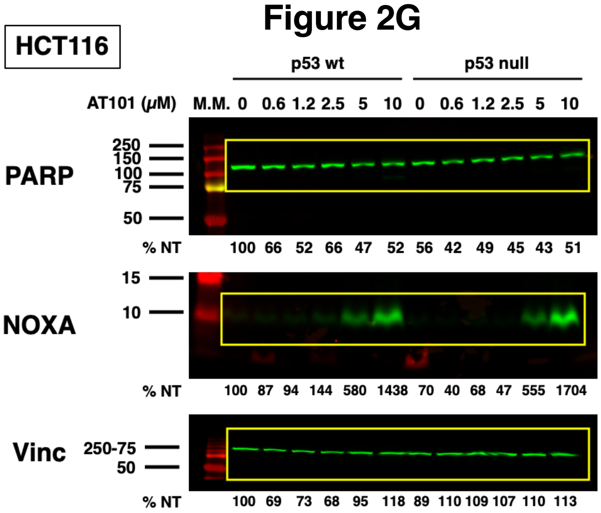

Figure 2I

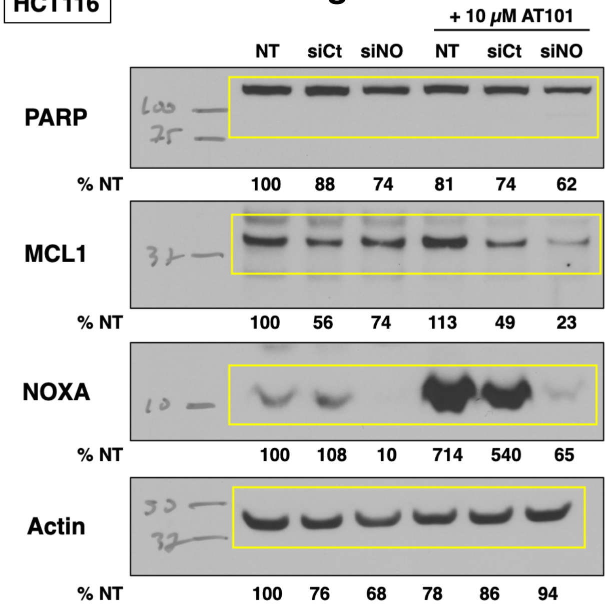

Figure 3A

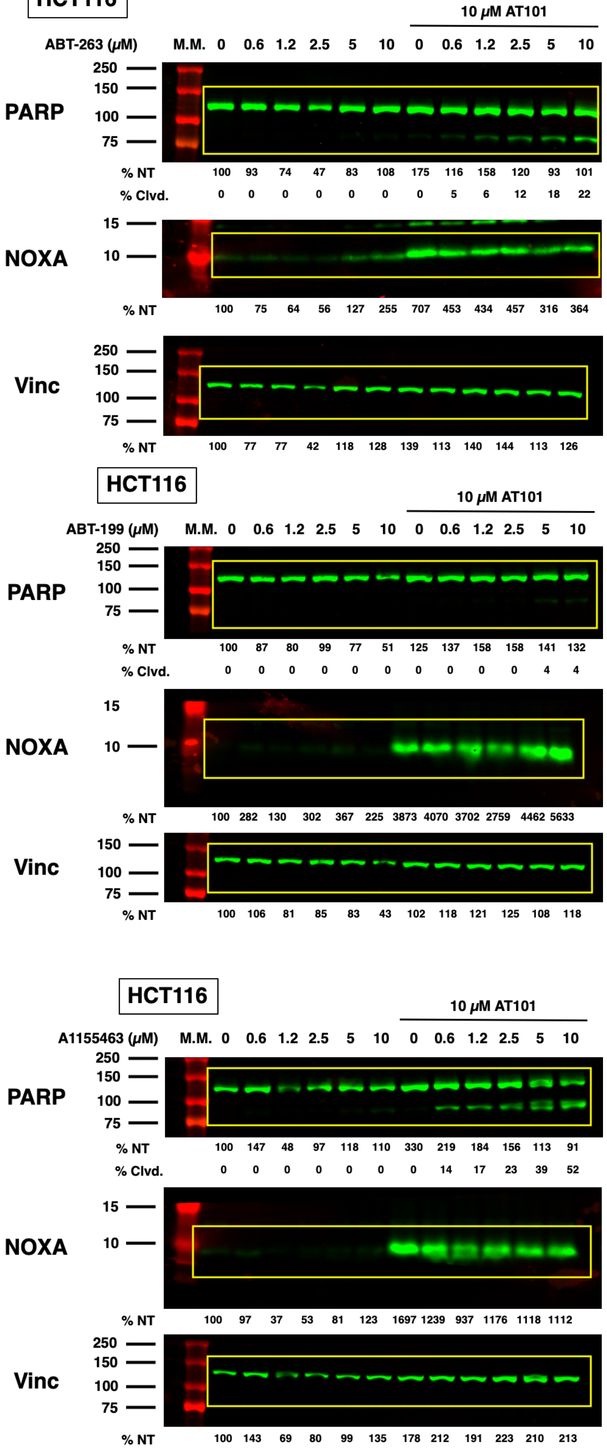

Figure 3B

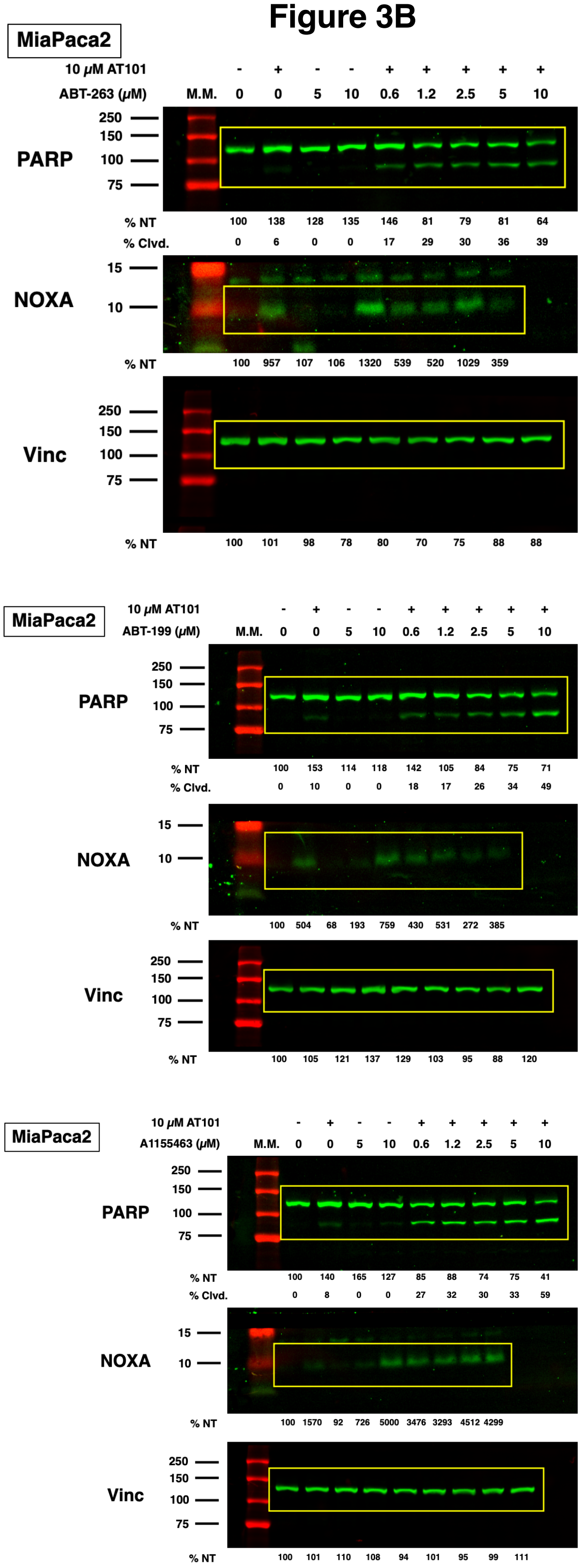

Figure 3C

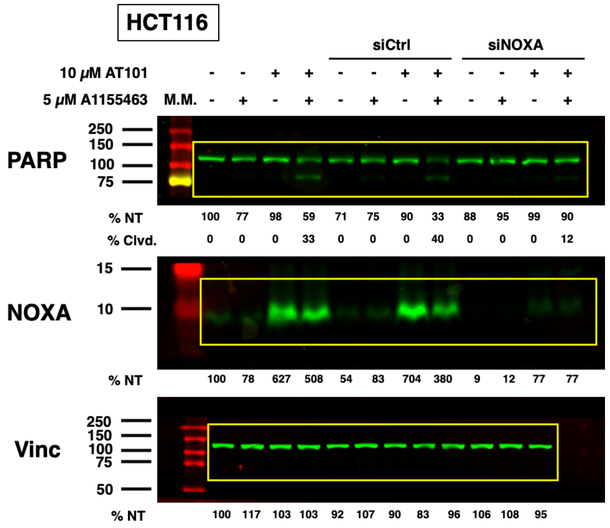

Figure 3D

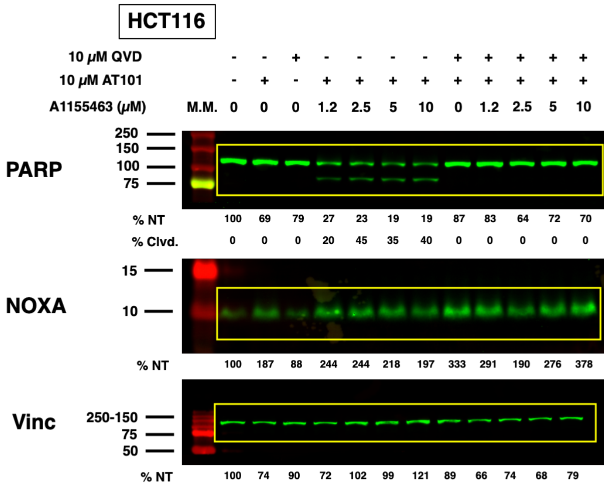

Figure 4A

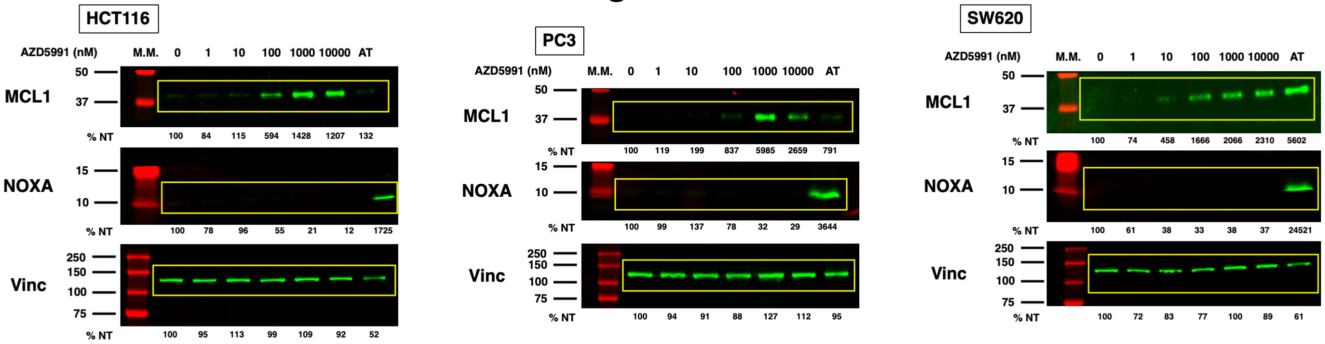

Figure 4B

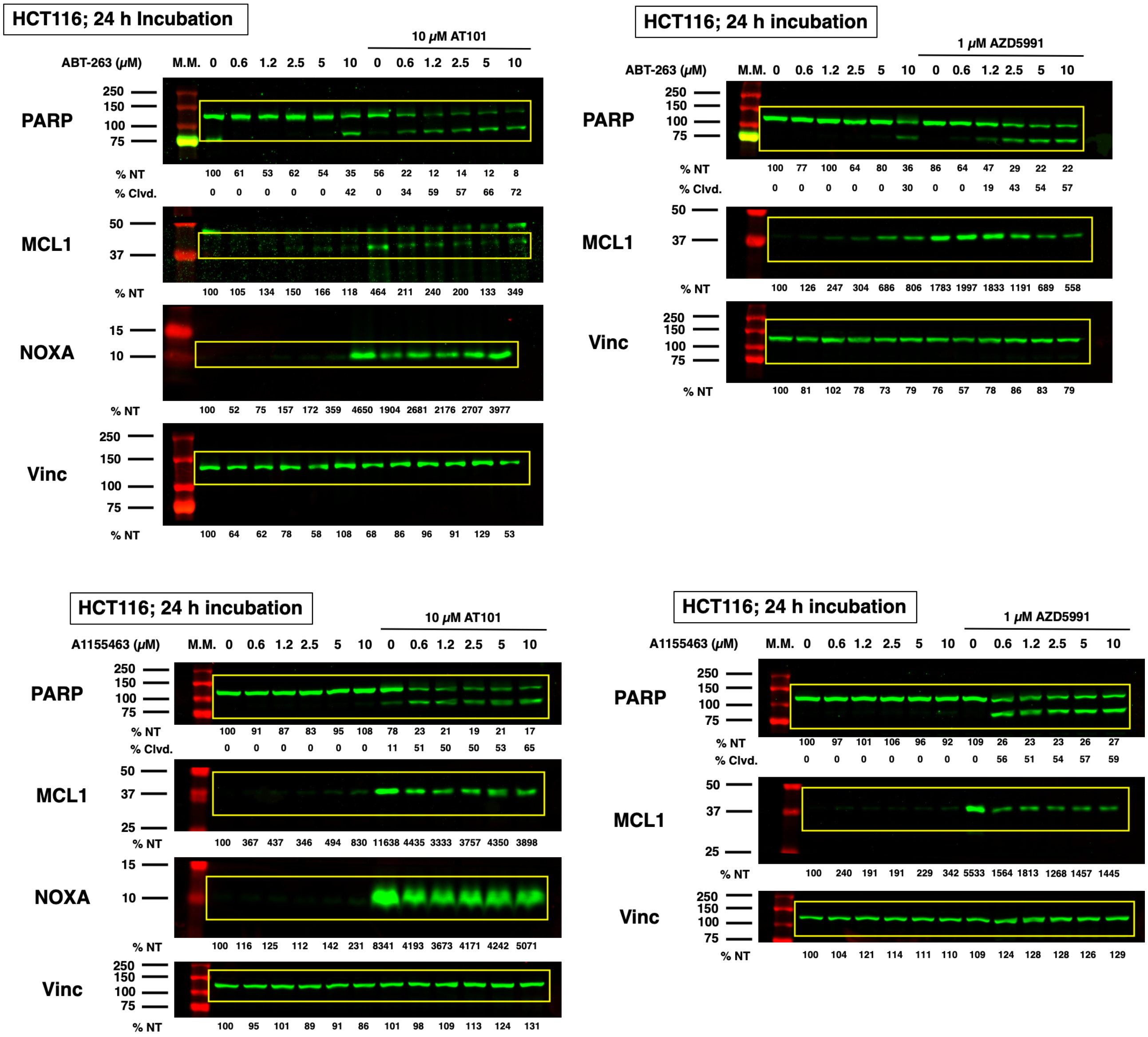

Figure 4C

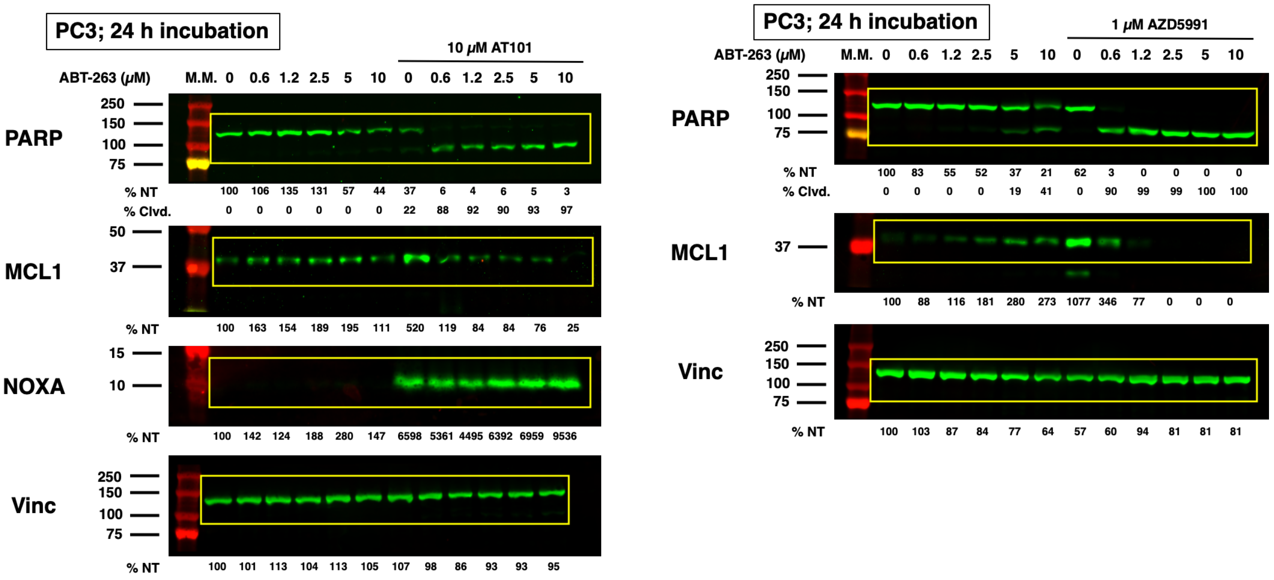

Figure 4D

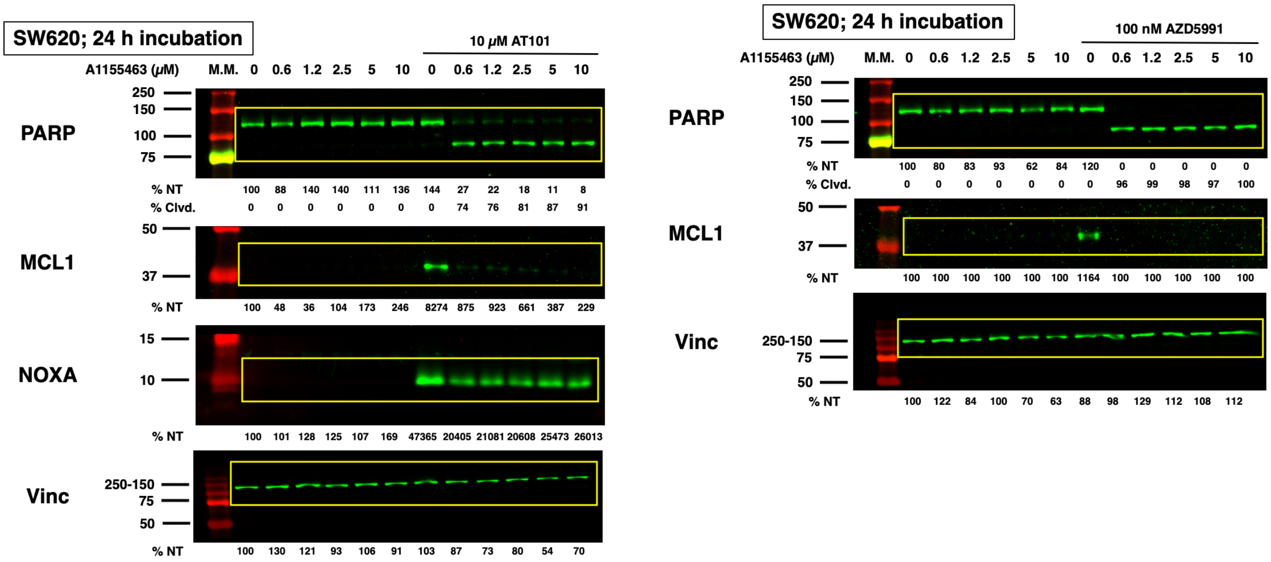

Figure 5A

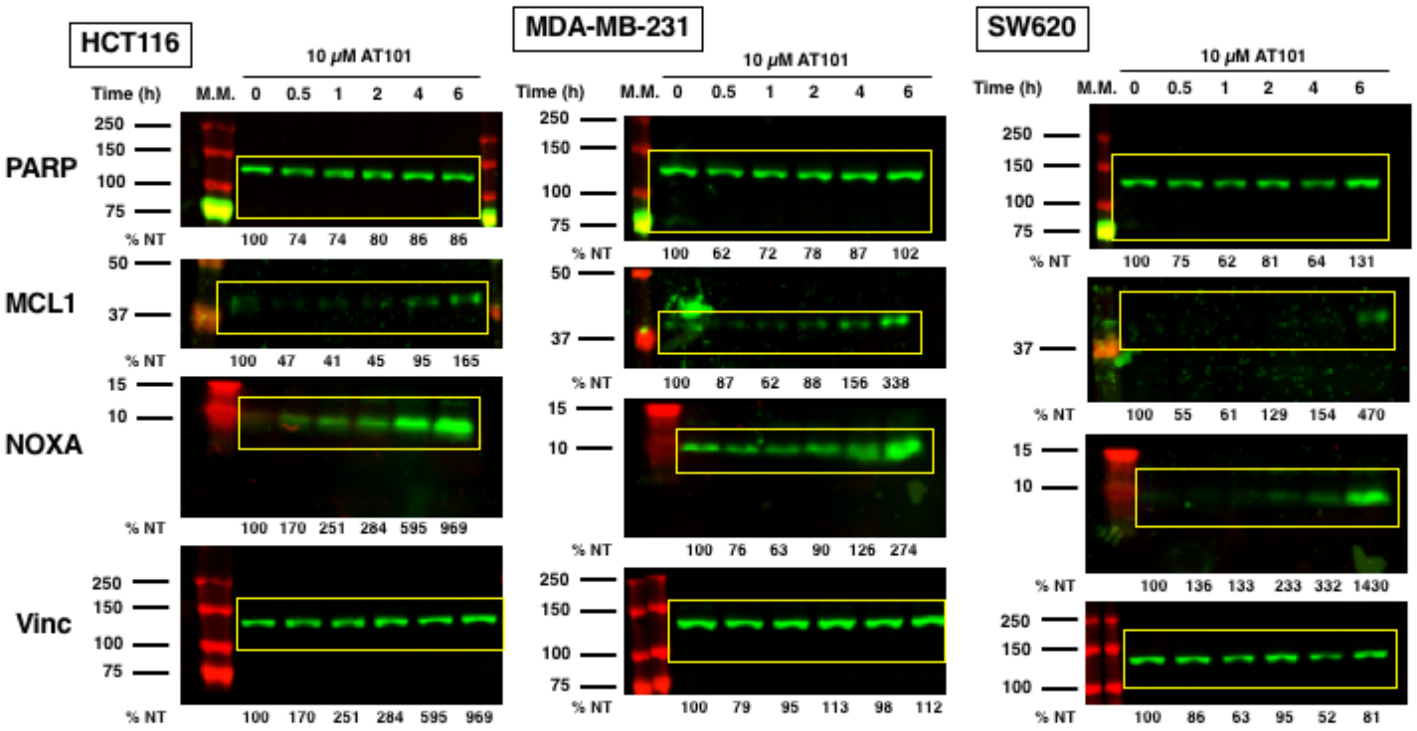

Figure 5B

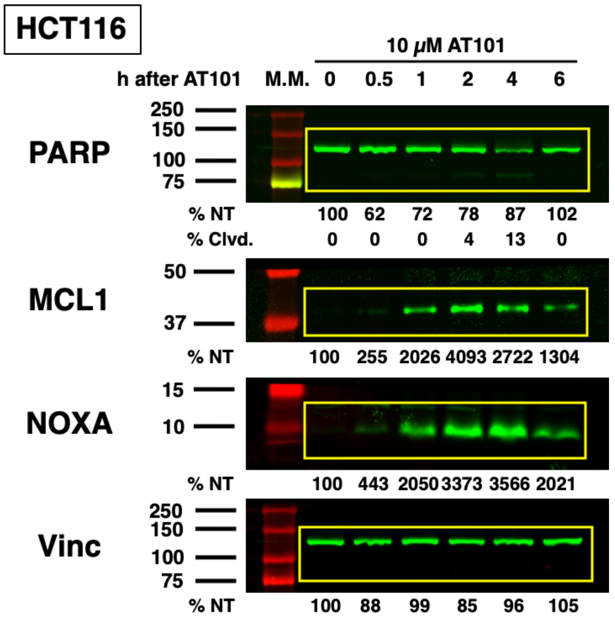

Figure 5C

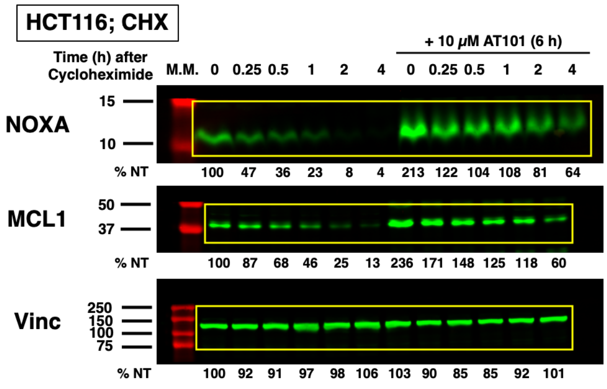

Figure 5D

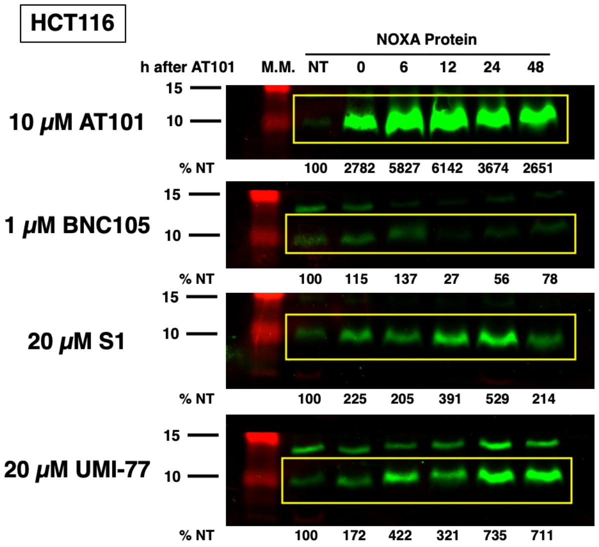

Figure 5E

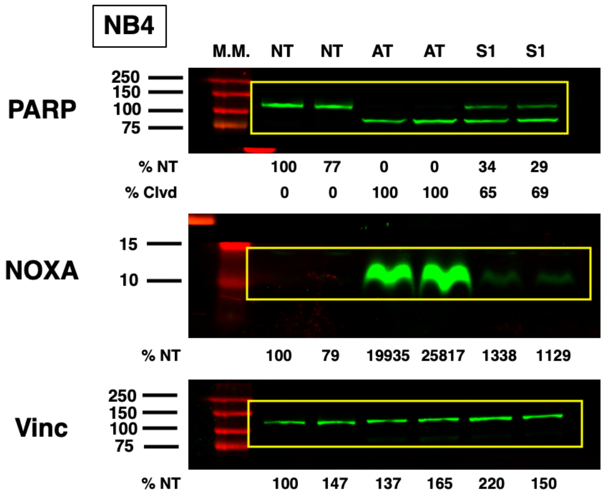

Figure 5F

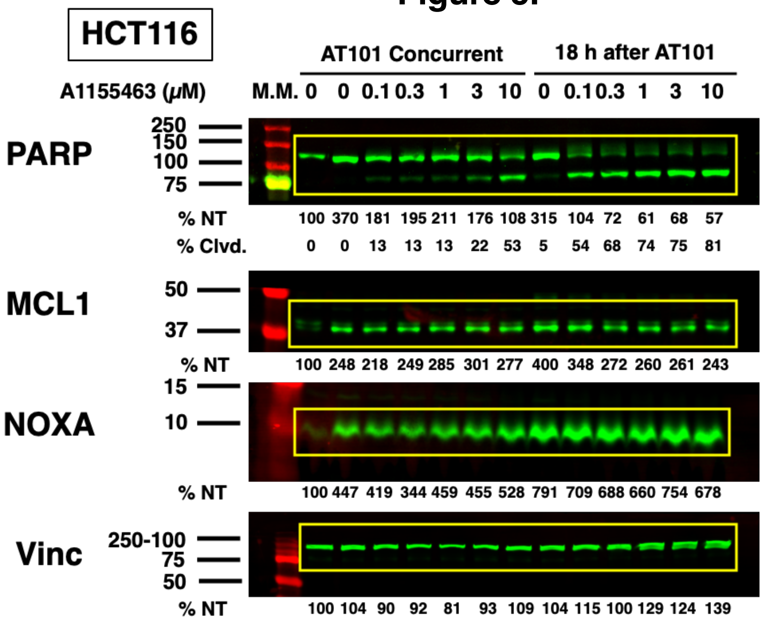

Figure 6A

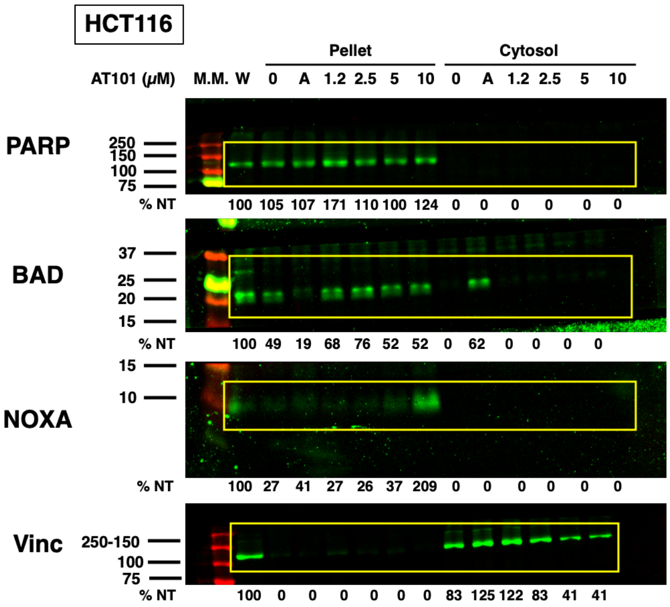

Figure 6B

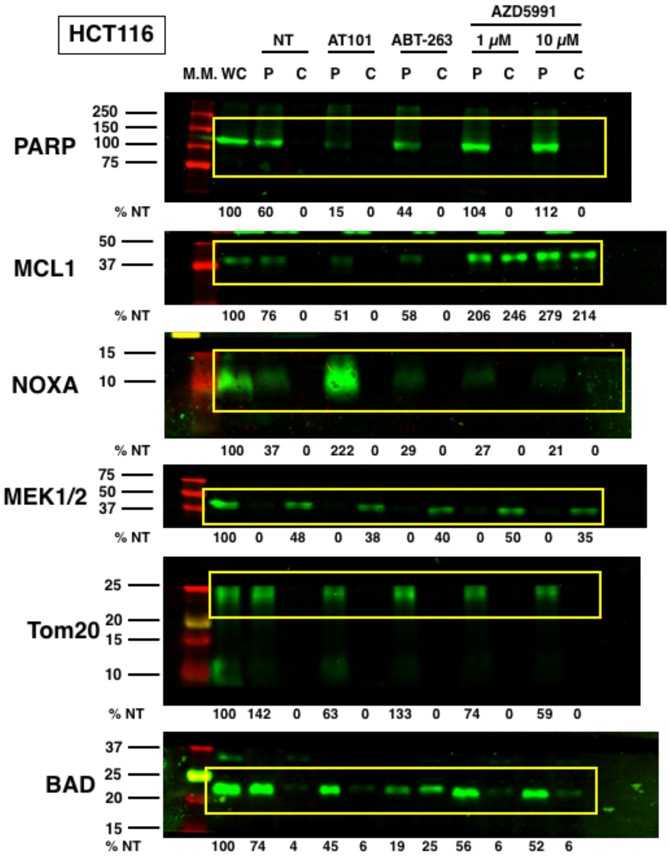

Figure 6C

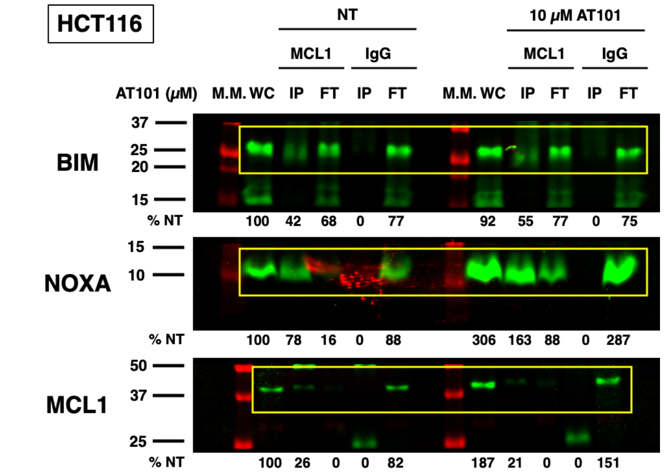

Supplement: Supplementary file 1 [file cancers-12-02298-s001.pdf]
